# Human Papillomavirus Prevalence in Urine Samples of Asymptomatic Male Sexual Partners of Women with Sexually Transmitted Diseases

**DOI:** 10.3390/ijerph182111706

**Published:** 2021-11-08

**Authors:** Hyunwoo Jin, Dong Hyeok Kim, Kyung Eun Lee

**Affiliations:** 1 Department of Clinical Laboratory Science, College of Health Sciences, Catholic University of Pusan, Busan 46252, Korea; jjinhw@cup.ac.kr (H.J.); ehd6091@naver.com (D.H.K.); 2 Clinical Trial Specialist Program for In Vitro Diagnostics, Brain Busan 21 Plus Program, The Graduate School, Catholic University of Pusan, Busan 46252, Korea

**Keywords:** male, human papillomavirus, urine, real-time PCR

## Abstract

Human papillomavirus (HPV) infection in males is associated with various cancers, including cervical cancer in women and penile and bladder cancers in men. However, there is limited research on the prevalence and prevention of male HPV infection. Moreover, a rapid test that can prevent the increase in HPV infection is needed. In this study, the prevalence of sexually transmitted pathogen (STP) and HPV infection was analyzed using real-time polymerase chain reaction assay in random urine samples collected from asymptomatic male sexual partners of women with sexually transmitted diseases. Among 130 men, 65 (50.0%) had STP and 12 (9.23%) had HPV infection. There was no association between STP and HPV infection. Among 12 cases of HPV infection, three were HPV-16 single infections, six were multiple infections, including HPV-16, and three of other high-risk HPV infections. Our results suggest the need for STP testing, including HPV testing, in sexual partners of high-risk women with sexually transmitted diseases, even in men without clinical symptoms (asymptomatic). Further research should be conducted by diversifying urine samples. We report the most convenient method for HPV detection, and it is expected to be widely applied to prevent sexually transmitted diseases in men and women.

## 1. Introduction

Human papillomavirus (HPV) is a sexually transmitted disease (STD) that is a major cause of cervical cancer in women. Recently, it has been reported that HPV is related to penile, anogenital, bladder, lung, and oropharyngeal cancers in men [1,2]. Although studies on the prevalence of HPV infection in men are insufficient compared to that in women, previous studies have reported that the prevalence of HPV infection is high in men who are sexual partners of women with an STD and women with human immunodeficiency virus infection [3]. In addition, the prevalence of HPV infection among men in Asia was reported to be lower than that among men in Africa, Europe, and the United States [4,5]. The prevalence of male HPV infection is reported differently depending on the region and ethnicity, but it is considered that there is a lack of research data on male HPV infection in Asia, including South Korea.

Men with HPV infection are often asymptomatic, which can increase the number of asymptomatic infections by them being a reservoir of HPV and an infection carrier [6]. Because HPV infection is transmitted by sexual activity, an increase in the prevalence of HPV infection in males may induce persistent HPV infection in female sexual partners, thus affecting the incidence of cervical cancer. Therefore, more research data are needed on the prevalence of HPV infection in men and the epidemiology of transmission of HPV infection to sexual partners.

In the case of sexually active males, the HPV infection rate is reported to vary widely, from 1.0% to 82.9% [7], which is interpreted to be due to differences in the anatomical location and sampling method [4]. The prevalence of HPV infection in asymptomatic men in China and the United States was 8.8% in genital samples, which is higher than that of 1.9% in oral samples [8,9]. However, the mechanism by which oral and genital infections occur simultaneously or whether infections occur sequentially is not clear. In another study, the prevalence of HPV infection in male genital organs, such as penile shaft, glans, and scrotum, was higher than that in urine samples [10]. However, to detect HPV infection in a penis specimen, cells must be collected by rubbing the entire glans penis, coronal sulcus, and foreskin using a cotton swab or brush [11]. Most asymptomatic men tend to be reluctant to undergo these cumbersome testing procedures, which makes it difficult to accurately determine the prevalence of HPV infection in men.

Considering the feasibility of using a sample for screening test, the urine specimen is a useful sample to test for the presence of male HPV infection; it can be easily obtained through a noninvasive method. A previous study reported high sensitivity for HPV DNA testing using urine samples [12], but another study reported inconsistent results [13]. Another study reported a higher prevalence of HPV infection in urine samples collected from men with urethritis than in those collected from healthy men [11].

In South Korea, women over the age of 20 years participate in a national screening system that allows free cervical cytology to be tested every two years, but there are no guidelines for the prevention of male HPV infection. There is also a lack of information on the anatomical location, sampling, and testing methods for male HPV testing, which limits the diversity of the research. It is therefore important to continuously investigate the prevalence of HPV infection in sexual partners of women with STD, including in men without clinical symptoms (asymptomatic). There is also an urgent need to develop a rapid testing method that can prevent the increase in HPV infection. In this study, we aimed to collect basic data on the epidemiology of transmission of HPV infection through sexual partners; therefore, the prevalence of HPV infection was determined using urine samples collected from asymptomatic men who are sexual partners of women with an STD.

## 2. Materials and Methods

### 2.1. Study Subjects

This study was conducted from January to October 2020; we collected random urine samples from an asymptomatic male sexual partner of a female patient diagnosed with STD at a women’s clinic in Busan. STD-positive female patients were defined as patients with positive cases of at least one of the 12 types of PANA RealTyper^TM^ STD Kit (PANAGENE, Daejeon, Korea), excluding the HPV test.

With the approval of the Institutional Ethics Committee of the Catholic University of Pusan (approval number: CUPIRB-2020-01-11), residual urine samples that could not be identified individually were used.

### 2.2. Sample Preparation

#### DNA Extraction Using Chelex

DNA was extracted using 5% Chelex^®^ 100 Resin solution (Bio-Rad, Hercules, CA, USA) according to the manufacturer’s instructions. The sample (200 μL) was transferred to a DNase-free tube and centrifuged at 8000× *g* for 5 min; the supernatant was removed, and 100 μL of 5% Chelex^®^ 100 Resin solution was added to the remaining cell pellet, followed by incubation at 100 ℃ for 10 min and centrifugation of the sample at 11,000× *g* for 10 min. The supernatant was transferred to a new DNase-free tube. The extracted genomic DNA was checked for concentration and purity using NanoDrop 2000 (Thermo Fisher Scientific, Wilmington, DE, USA) and stored at a freezing temperature of −20 °C before analysis.

### 2.3. Sexually Transmitted Pathogen Detection

Detection of 12 species of pathogens (Chlamydia trachomatis, Ureaplasma parvum, Trichomonas vaginalis, Mycoplasma genitalium, Mycoplasma hominis, Neisseria gonorrhoeae, Ureaplasma urealyticum, Gardnerella vaginalis, Candida albicans, Treponema pallidum, Herpes simplex virus Ⅰ), and Herpes simplex virus Ⅱ) that cause STDs was performed by real-time polymerase chain reaction (PCR) according to the instructions in the PANA RealTyper™ STD Kit.

#### PANA RealTyper™ STD Kit

Real-time PCR was performed using the CFX96 Touch™ Real-Time PCR Detection System (Bio-Rad, Hercules, CA, USA), and the species were detected by melting curve analysis. For real-time PCR, 19 µL of STD Mix was mixed with 1 µL of Taq DNA polymerase; then the mixture was combined with 5 µL of the extracted DNA to make a total volume of 25 µL, and then real-time PCR assay was performed. After real-time PCR assay, the baseline value for each fluorescence dye in the STD mix was set, and the infection and genotype were determined according to the melting temperature value of the melting peak for each fluorescence signal.

### 2.4. HPV Detection

HPV detection was analyzed by real-time PCR assay using the MolecuTech Real HPV 16/18/HR^®^ Kit (YD, YOUNGIN, South Korea). The primer/probe mixer contained 14 high-risk HPV (HR-HPV) and β-globin-specific primers and probes. The amplified DNA was detected using four different fluorescently labeled probes (FAM; HPV type-16, LC RED 610; HPV type-18, HEX; HR-HPV, CY5: internal control). HPV types 16, 18, and β-globin signals are detected with different fluorescent materials, and 12 high-risk groups (31, 33, 35, 39, 45, 51, 52, 56, 58, 59, 66, and 68) were detected with the same fluorescent material.

#### MolecuTech Real HPV 16/18/HR^®^ Kit

Real-time PCR assay was performed using the CFX96 Touch™ Real-Time PCR Detection System (Bio-Rad, Hercules, CA, USA), and real-time PCR conditions included a pre-denaturation step for 3 min at 94 °C; three cycles of denaturation for 20 s at 94 °C, annealing for 40 s at 48 °C, and elongation for 40 s at 72 °C. The subsequent 40 cycles were 20 s at 94 °C, 40 s at 52 °C, and 40 s at 72 °C. The Ct values of the positive judgment criteria were as follows: FAM (HPV type 16) had a Ct value of <34, LC Red 610 (HPV type 18) had a Ct value of <40, HEX (HR-HPV) had a Ct value of <34, and CY5 (internal control) had a Ct value of <38.

HPV DNA sequencing was performed by nested PCR using MY09/MY11 primers for the first amplification and GP5/GP6 primers for the second amplification. Each PCR product was analyzed by electrophoresis on a 2% agarose gel. HPV-infected samples were detected by electrophoresis at 150 bp. Samples were amplified near 150 bp and extracted using the gel extraction method. The nested PCR products were re-amplified using the GP5 primer with BigDye Terminator (Applied Biosystems, Waltham, CA, USA) and then subjected to HPV DNA sequencing using an automatic DNA sequencer, ABI3730XL (Applied Biosystems, Waltham, CA, USA). Identity analysis was performed to confirm the HPV genotype using the Basic Local Alignment Search Tool (BLAST), version 2.10.1 (https://blast.ncbi.nlm.nih.gov/, accessed on 14 July 2021).

### 2.5. Statistical Analysis

Statistical analysis was performed using the Statistical Package for the Social Sciences (SPSS) for Windows Standard, version 19.0 (SPSS, Chicago, IL, USA). Fisher’s exact test was used to analyze the correlation between the prevalence of HPV infection and a STP-positive result in random urine samples of asymptomatic men. Statistical significance was set at *p* < 0.05.

## 3. Results

Among the sexual partners of STD-positive women, a total of 130 men visited the hospital for complete treatment, and none of them had clinical symptoms. The average age of asymptomatic men was 37.7 years, 19 in their 20s, 66 in their 30s, 33 in their 40s, 9 in their 50s, and 3 in their 60s. Sexually transmitted pathogen (STP) detection showed a positive result in 65 patients (50.0%) out of 130 random urine samples of men. Among them, 43 cases were single infection and 42 cases were multiple infections. Among the 12 types of STDs, *Gardnerella vaginalis* infection was the most common (48.2%, 41/85), followed by *Ureaplasma urealyticum* infection (29.4%, 25/85). *Ureaplasma parvum* infection occurred in 9.4% of patients (8/85), *Chlamydia trachomatis* infection occurred in 5.9% (5/85), *Mycoplasma hominis* infection occurred in 5.9% (5/85), and *Mycoplasma genitalium* infection occurred in 1.2% (1/85) (Table 1).

The average age of HPV-positive men was 38.4 y (range, 32–53 y). HPV detection showed a positive result in 12 (9.23%) of 130 random urine samples from men. Among them, there were three cases of HPV-16 single infection and six cases of multiple infections, including HPV-16, and three cases of other high-risk HPV infections. According to the results of HPV DNA sequencing analysis of 12 urine samples in which HPV infection was diagnosed by real-time PCR assay, the presence of HPV-16 genotype was confirmed in two of three cases of HPV-16 single infection. In multiple infections including HPV-16, no genotype was confirmed in any of the six cases, and in the other HR-HPV cases, two cases of the HPV-35 genotype and one case of the HPV-33 genotype were confirmed (Table 2).

Regarding the correlation between HPV infection and STP infection, eight patients (8/12, 66.7%) were HPV-positive for an STP-positive result, and four (4/12, 33.3%) were HPV-positive for an STP-negative result. This result was not statistically significant (Table 3).

## 4. Discussion

Because HPV is an STD, the prevalence of HPV infection is expected to be similar in men and women, but the prevalence of HPV infection in cervical cancer is reported to be much higher than that in penile cancer [1,2,14]. Although the exact mechanism underlying this has not been elucidated, it has been reported that the transformation zone of the squamous–columnar junction, where cervical intraepithelial tumors frequently occur, is susceptible to HPV infection. However, there are reports of a lower prevalence of HPV infection in men than in women because the urethra and penis are covered with squamous epithelial tissue and there is no transformation zone, as in women [14]. It is also known that oncogenic HPV infection in men with penile intraepithelial tumors is highly correlated with cervical intraepithelial tumors in their female partners [14,15,16]. This suggests that HPV infection can be transmitted through sexual intercourse, and if men have high-risk HPV infection without clinical symptoms (asymptomatic), then it is thought to have an adverse effect on the prevention of cervical cancer in women. In this study, sexual partners were tested to completely treat women with an STD, but most men refused it because they had no clinical symptoms, and all 130 men who underwent the test were asymptomatic.

An analysis of the prevalence of HPV infection in the penis, urethra, and urine samples showed that the penis sample had the highest prevalence and the urine sample showed the lowest [3]. Therefore, it has been reported that samples collected from the external region of the male genital tract show a higher HPV detection rate [11]. However, because most men without clinical symptoms do not consent to undergo testing of penile and urethral samples, there is a concern about the continued infection of STD, including HPV infection.

Urine samples are considered the easiest and most convenient screening sample for male patients with HPV. In Argentina, the usefulness of using a urine sample was highly evaluated, reporting that the first voided urine sample of men with penile lesions or a HPV-positive sexual partner showed high HPV DNA (73%) [17]. Another study reported that the prevalence of HPV infection was as high as 50% in urine samples collected from patients with prostate cancer [18]. However, there have been studies explaining that the transport of viruses in male penile lesions, such as HPV-6 or HPV-11, to the urethra is abnormal [19]. In another study, high-risk HPV infection was shown to be transmitted to the terminal urethra through sexual contact, and the virus migrated through the urethra to the urinary tract epithelium of the bladder, leading to bladder cancer [20]. In addition, because the detection rate of HPV-16 in urethral samples was higher than that in penile samples, the importance of using urethral samples in addition to penile samples was emphasized to monitor HPV infection [3,20]. As such, the prevalence of HPV infection is reported differently depending on the sample used according to the male anatomy; therefore, it is thought that a manual/guideline for managing this infection is needed. In this study, HPV detection was positive in 12 (9.23%) of 130 random urine samples. Among them, three cases were single infection with HPV-16, six cases were multiple infections, including HPV-16 and three cases of other high-risk HPV infections. According to the results of HPV DNA sequencing analysis of 12 urine samples in which HPV infection was diagnosed by real-time PCR assay, the HPV-16 genotype was confirmed in two out of three cases of single infection with HPV-16. In multiple infections including HPV-16, no genotype was confirmed in any of the six cases, and in the other HR-HPV cases, two cases with the HPV-35 genotype and one case with the HPV-33 genotype were confirmed. Sequencing analysis has a limitation in terms of not confirming multiple infections, but infection including HPV-16, which is the main cause of cervical cancer, accounted for nine out of 130 (6.92%) urine samples collected from asymptomatic men; therefore, the epidemiology of infection transmission through sexual partners should be of interest.

In this study, random urine samples were convenient, as they were obtained through an easy method from asymptomatic men who were indirectly exposed to an STD. However, there are no appropriate guidelines for collecting urine samples. Another study reported that the use of the first voided urine sample in a high-risk male cohort was useful for HPV DNA detection and genotyping [17]. In particular, the importance of testing first-voided urine and quantifying viral load level was reported [21,22], an extraction method with a sufficient amount of whole urine and preservatives was also proposed [23,24]. Additionally, the detection rate of HPV improved when the urine sample was stored overnight at 4 °C [25]. In addition, studies have reported the usefulness of the HPV test method for testing urine samples using liquid-based cytology-preserving solution, which is used for the screening for female uterine cancer [26]. However, the detection of HPV in urine samples suggests that the virus can infect all parts of the urinary tract, such as the urethra, prostate, and bladder, in men; therefore, it should be carefully observed. Therefore, it is necessary to develop an easy and simple testing method to apply the HPV test for general asymptomatic men, and urine samples are expected to be the key to solving this problem.

In recent studies on HPV infection, it was reported that the HPV infection rate was related to infection with other STPs, such as Neisseria gonorrhoeae and Chlamydia trachomatis [27], and the incidence of HPV infection in particular was rapidly increasing among young people [4]. In this study, STP infection was found to be 50.0% (65/130), and there was no statistical correlation between STD and HPV infection, but follow-up is needed to determine whether it is the cause of persistent HPV infection. In this study, the average age of HPV-positive men was 38.4 years, and the study subjects also included 19 men in their 20s and 66 men in their 30s who were indirectly exposed to an STD, which is considered a case reflecting recent research results. Therefore, if HPV infection in young asymptomatic men is maintained as a persistent infection for a long time, then it may be a risk factor for the health of sexual partners; therefore, early management is required.

HPV infection in women is the main cause of cervical cancer, and as it has become commonly known among the general public, the rate of using classical Papanicolaou test or HPV DNA test for confirming HPV infection is increasing over time, which makes it possible to diagnose and manage HPV infection at an early stage. The national screening manual to prevent the risk of HPV infection in men is not properly prepared, and this needs to be urgently improved if it reflects the trend that the sexually active population includes the younger individuals.

This study has limitations. To determine the prevalence of HPV infection in random urine samples collected from asymptomatic men indirectly exposed to an STD in this study, the study was limited to sexual partners of STD-infected women; thus, the number of study subjects was not large. In addition, we could not analyze the correlation of HPV infection with sexual partners. In the future, more research is needed to determine the types of urine samples suitable for diagnosing HPV, such as first-voided urine, random urine, and liquid-based cytologic urine in men. It is also considered that prospective research methods to identify the route of infection with sexual partners should be considered.

## 5. Conclusions

Urine-based testing is the most convenient method and is thought to be widely used in preventing the incidence of STD in men and women. In addition, even if asymptomatic, it is considered that treatment for male or female sexual partners should be performed at the same time as the epidemiological route of sexually transmitted infections.

## Figures and Tables

**Table 1:** PANA RealTyper™ STD-positive results in random urine samples of men.

Sexually Transmitted Pathogens	Infection		
	Single	Multiple	Total
*Chlamydia trachomatis*	3 (7.0)	2 (4.8)	5 (5.9)
*Ureaplasma urealyticum*	11 (25.6)	14 (33.3)	25 (29.4)
*Ureaplasma parvum*	5 (11.6)	3 (7.1)	8 (9.4)
*Mycoplasma genitalium*	1 (2.3)	-	1 (1.2)
*Mycoplasma hominis*	-	5 (11.9)	5 (5.9)
*Gardnerella vaginalis*	23 (53.5)	18 (42.9)	41 (48.2)
Total	43 (100.0)	42 (100.0)	85 (100.0)

STD, sexually transmitted disease.

**Table 2:** HPV sequencing results from 12 male urine samples diagnosed by real-time PCR HPV DNA test.

	Infection	Sequencing		Genotype
		Detection	Undetection	
Real time-PCR	HPV-16 (single)	2	1	16
	HPV-16 (multiple)	-	6	-
	Other HR-HPV	3	-	33, 35, 35

PCR, polymerase chain reaction; HPV, human papillomavirus.

**Table 3:** Association between the prevalence of HPV infection and an STP-positive result in male urine samples.

Infection		STP		Total	P
		Positive	Negative		
HPV	Positive	8	4	12	NS
	Negative	57	61	118	
Total		65	65	130	

HPV, human papilloma virus; STP, sexually transmitted pathogen; NS; not significant.
